# The Healthy Infant Nasal Transcriptome: A Benchmark Study

**DOI:** 10.1038/srep33994

**Published:** 2016-09-23

**Authors:** Chin-Yi Chu, Xing Qiu, Lu Wang, Soumyaroop Bhattacharya, Gerry Lofthus, Anthony Corbett, Jeanne Holden-Wiltse, Alex Grier, Brenda Tesini, Steven R. Gill, Ann R. Falsey, Mary T. Caserta, Edward E. Walsh, Thomas J. Mariani

**Affiliations:** 1 Division of Neonatology and Pediatric Molecular and Personalized Medicine Program, University of Rochester Medical Center, Rochester NY, USA; 2 Department of Biostatistics and Computational Biology, University of Rochester Medical Center, Rochester NY, USA; 3 Department of Medicine, University of Rochester Medical Center, Rochester NY, USA; 4 Department of Microbiology and Immunology and University of Rochester Medical Center, Rochester NY, USA; 5 Division of Pediatric Infectious Diseases, University of Rochester Medical Center, Rochester NY, USA; 6 Department of Medicine, Rochester General Hospital University of Rochester Medical Center, Rochester NY, USA

## Abstract

Responses by resident cells are likely to play a key role in determining the severity of respiratory disease. However, sampling of the airways poses a significant challenge, particularly in infants and children. Here, we report a reliable method for obtaining nasal epithelial cell RNA from infants for genome-wide transcriptomic analysis, and describe baseline expression characteristics in an asymptomatic cohort. Nasal epithelial cells were collected by brushing of the inferior turbinates, and gene expression was interrogated by RNA-seq analysis. Reliable recovery of RNA occurred in the absence of adverse events. We observed high expression of epithelial cell markers and similarity to the transcriptome for intrapulmonary airway epithelial cells. We identified genes displaying low and high expression variability, both inherently, and in response to environmental exposures. The greatest gene expression differences in this asymptomatic cohort were associated with the presence of known pathogenic viruses and/or bacteria. Robust bacteria-associated gene expression patterns were significantly associated with the presence of *Moraxella*. In summary, we have developed a reliable method for interrogating the infant airway transcriptome by sampling the nasal epithelium. Our data demonstrates both the fidelity and feasibility of our methodology, and describes normal gene expression and variation within a healthy infant cohort.

Sampling of the airway for research or diagnostic biospecimens in diseased infants is highly challenging. As compared to the tremendous progress made over the last decades in our understanding of adult lung diseases such as COPD and lung fibrosis^1^,^2^,^3^, this limitation has restricted progress in identifying the molecular mechanisms contributing to pediatric respiratory diseases. Many contemporary studies sample the upper airway, using aspirates of tracheal secretions in intubated subjects to collect intra-airway inflammatory cells and sloughed resident cells of the airway. The availability of these samples is limited to intubated subjects, introducing significant selection bias. Furthermore, the ability of these samples to reliably capture the cellular and molecular state of the lung is unclear. Other studies have analyzed nasal washings, which can be performed in a more broad population. Again, these samples are of unclear relevance to the pathophysiology of the lung.

Brushings of nasal epithelial cells have been used for decades to diagnose primary ciliary dyskenesis (PCD), indicating that recovery of epithelial cells from the nose of non-acutely ill children and infants could provide molecular information that reflects abnormalities of physiological relevance in the distal airway. Furthermore, nasal brushings in adults and older pediatric patients have previously been used for interrogation of the transcriptome. These studies have generally shown that nasal brushings broadly capture gene expression with relevance to the airways^4^, and can detect disease-associated changes in gene expression^5^.

We undertook the present study to determine if the recovery of nasal epithelial cells for transcriptomic analysis was feasible, and whether these data could provide molecular information of relevance to the more distal airways. We present a reliable procedure for sampling resident cells of the airway in healthy young infants, which is applicable to infants experiencing respiratory infectious disease (T. Mariani, unpublished observations), and likely to older ill and healthy pediatric subjects. Furthermore, we provide a benchmark healthy infant nasal transcriptome (HINT) data set, describing baseline expression and natural variation, for asymptomatic subjects. Our data demonstrates that it is technically feasible to quantify genome-wide RNA expression from young infant’s airways, which may be helpful in the evaluation of host responses to diseased states.

## Results

### Subject Demographics and RNA-Seq Data Generation

Fifty-three healthy, full-term infants were recruited at birth. A majority of the subjects were female, predominantly Caucasian, and had a mean gestational age of 39.3 ± 1.0 (mean ± SD) weeks, with a mean birth weight of 3.3 ± 0.4 kg (Table 1). Nasal brushing samples were obtained from the inferior nasal turbinate at approximately one month of age (see *Methods*). All samples were collected when infants were free of illness, specifically signs of respiratory infection or fever.

**Figure 1:**
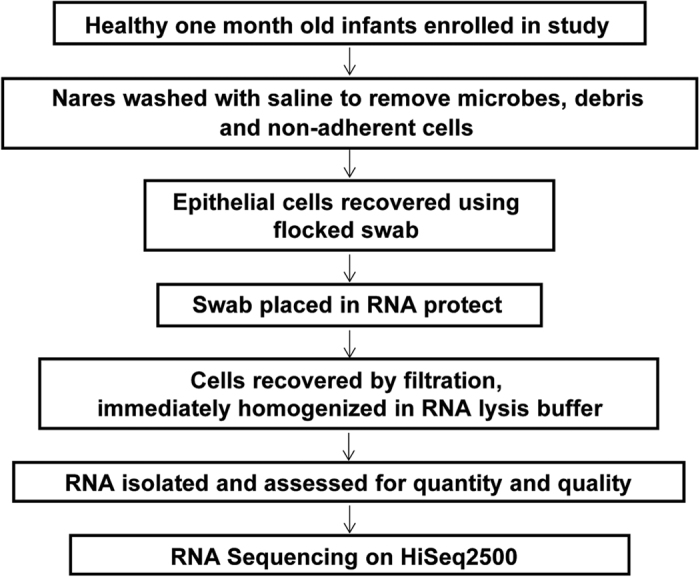
Nasal sample collection and transcriptome generation overview diagram. Summary of the major steps involved in healthy infant nasal airway sample collection and transcriptome generation. The procedure is described in Methods, and a detailed protocol is provided in the data supplement.

**Figure 2:**
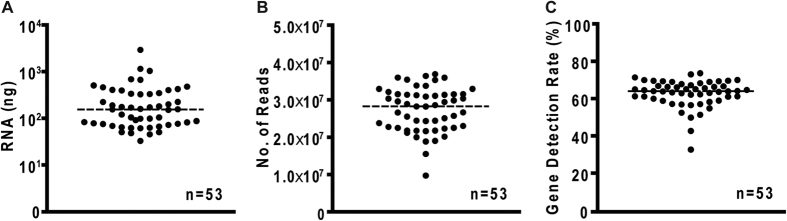
RNA and sequencing quality assessment. Details are provided for RNA recovered (**A**), total number of sequencing reads generated (**B**), and the proportion of the genome for which transcripts were detected (**C**), for each of the samples.

RNA was purified for high throughput sequence analysis from the recovered nasal cells as outlined in Fig. 1. RNA yields from the nasal brushings were variable with a mean recovery of 278.5 ± 436.7 ng (Fig. 2). Due to variability in total RNA recovery, cDNA was generated from approximately 1 ng input RNA. As shown in Fig. 2, the samples generated an average of 27 ± 6 million total reads, with 66 ± 14% displaying unique mapping to the human genome. After excluding genes whose expression was not robustly and reliably detected across subjects (see *Methods*), 13,978 genes or 63 ± 7% of the genome was transcriptionally represented in these samples.

### Cell-Specific Marker Gene Analysis

**Figure 3:**
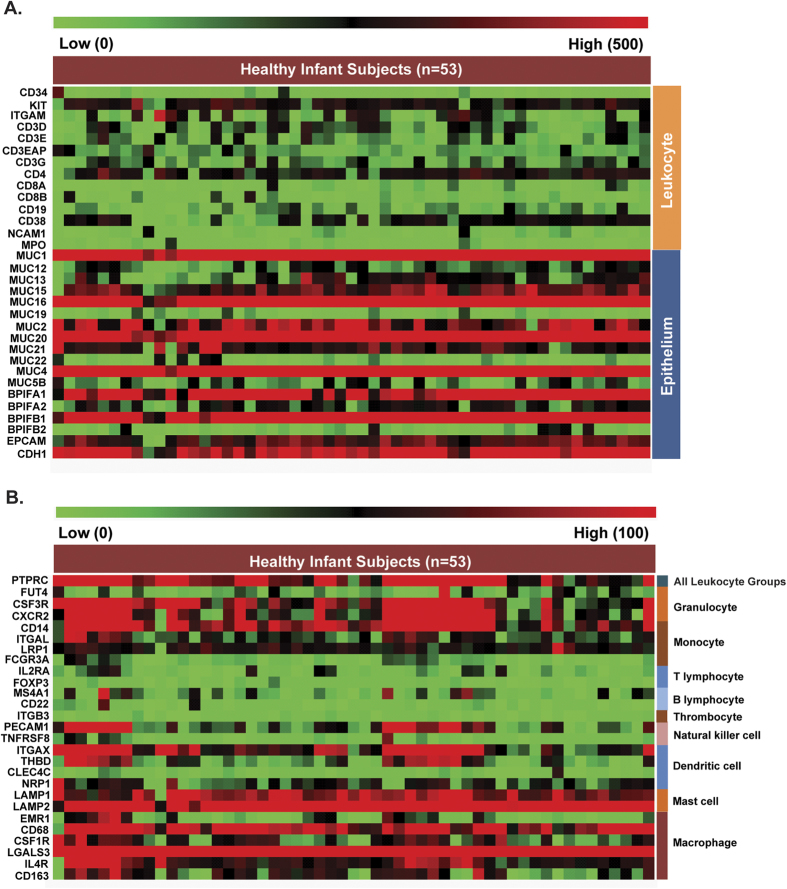
Candidate epithelial and inflammatory cell gene expression. Individual genes are indicated as rows, and individual subjects as columns. Relatively high expression is indicated by red and relatively low expression is indicated by green. (**A**) Expression estimates of candidate epithelial cell and leukocyte genes are presented. The data indicate consistently high levels of expression for epithelial cell markers including prototypical nasal epithelial cell genes (BPIFs), general epithelial cell genes (CDH1) and some mucosal epithelial cell markers (MUC1/2/4/16/20). Conversely, the data indicate relatively low levels of expression for most leukocyte markers including lymphocyte genes (CD3/8/19/56), neutrophil genes (MPO) and generic hematopoietic genes (CD34). (**B**) We further assessed the expression of a subset of genes used to define leukocyte subtypes. The data indicate that all leukocyte markers are expressed at a much lower level than epithelial cell markers. Among the leukocyte markers, genes associated with granulocytes, mast cells and macrophages appear to be most highly expressed.

We first interrogated the expression of cell type-specific marker gene expression in this data set, in order to assess the nature and composition of the samples (Fig. 3). We noted high levels of expression (>1,000 normalized counts) for transcripts for PLUNC, the definitive marker of nasal epithelial cells^6^. BPIFA1 (SPLUNC) and BPIFB1 (LPLUNC) were most highly expressed, as compared to their alternate isoforms. We also observed a high level of expression for other general epithelial cell markers, including CDH1 and EPCAM, and diverse expression of epithelial-specific mucins (MUC1/2/4/16/20).

We noted expression of some non-epithelial genes, but at lower expression levels (<100 normalized counts), so we thoroughly assessed the data set for evidence of non-resident cells. First, we assessed globin gene transcripts as a measure of red blood cells. The total burden of transcripts encoding any of the globin genes was <0.01%, with 29 (55%) of the subjects having zero reads mapping to any globin gene. These data indicate that bleeding in general, or contamination of samples with blood-derived cells, was not confounding our gene expression assessments. We next assessed the expression of selected lymphocyte and other immune cell markers. Most of these genes (e.g., CD3/8/19, MPO) were expressed at low levels in most subjects. However, the expression of some (e.g., CD4) were reliably detected in many or most subjects, albeit at a relatively low level. Finally, we assessed the expression of a set of immune cell marker genes^7^. These data suggested the presence of a low level of immune cells, primarily granulocytes, mast cells and macrophages (Fig. 3). However, it is clear that some subjects displayed higher levels of expression for leukocyte markers than others.

### The Infant Nasal Transcriptome

**Figure 4:**
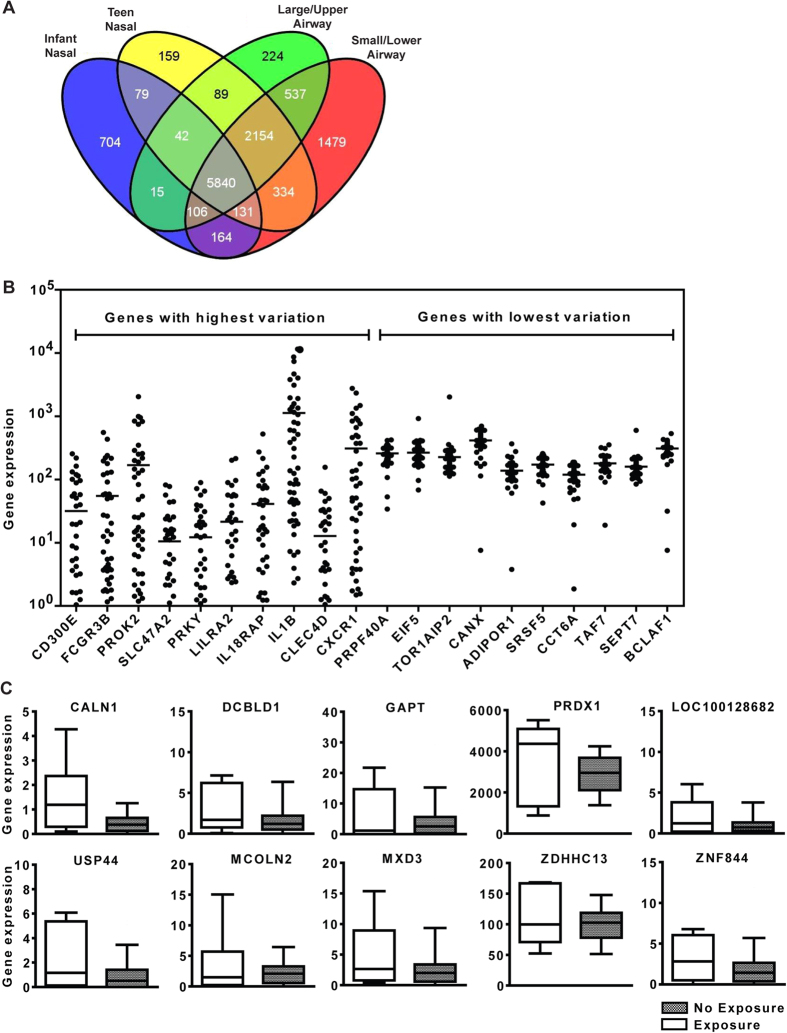
Analysis of human infant airway transcriptome (HINT). (**A**) We compared the 7081 non-ubiquitously expressed genes detected in the healthy infant nasal transcriptome, to those genes identified as representing airway transcritpomes in teenage nasal brushing, large/upper airway brushings and small/lower airway brushings. A large proportion of the genes (82%) were detected in both nasal, large and small airways. (**B,C**) We identified genes that displayed extremes of variation across subjects. (**B**) We calculated the coefficient of variation for expression of each gene across all subjects. Displayed are the relative expression estimates for the 10 genes with either the highest or lowest variation. (**C**) We tested for genes whose expression variance was significantly associated with all demographic variables. Displayed are the group-wise expression levels and variation in expression for 10 genes that displayed significant differences in expression variance in subjects exposed to environmental tobacco smoke as compared to those with no exposure.

To comprehensively define the transcriptome of the healthy infant nasal airway, we considered the 13,978 genes detected in our data set, and disregarded genes displaying ubiquitous expression across other tissues and samples^8^. This resulted in a set of 7,081 genes whose expression we propose defines the healthy infant nasal transcriptome (HINT). We compared the HINT to the nasal transcriptome previously reported for non-asthmatic control teenage subjects^4^. There was a high degree of similarity between these two transcriptomes (Fig. 4A), with the older subjects displaying more diversity than the infants. Since we believe that interrogation of the nasal transcriptome may provide insight into homeostatic and disease processes occurring in the lung proper, we compared the HINT to previously published data sets describing gene expression in the pulmonary upper^9^ or lower^10^ airways. Among the 7,081 expressed genes defining the HINT, 5840 (82%) were expressed in all samples, while 86% were expressed in “teenage” nasal airway, and 88% were consistently expressed in the adult (both upper and lower) airway.

We next assessed global HINT expression in an unbiased manner. We found the nasal and upper airway specific epithelial cell markers BPIFA1/SPLUNC1 and BPIFB1/LPLUNC1, were the 11^th^ and 12^th^ most highly expressed transcripts based upon mean expression level, more highly expressed than ACTB (rank 20) and GAPDH (rank 92). Among the other most highly expressed transcripts in the HINT were for MUC-16 (rank 35), −1 (rank 48), −4 (rank 73), −20 (rank 76), and two epithelial-specific keratins (KRT 19/rank 53 and KRT8/rank 129). Other highly expressed transcripts included B2M (rank 2) which encodes the antimicrobial b-2-microglobulin, SAT1 (rank 3) which encodes a regulator of intracellular polyamines concentration, FTL (rank 4) which encodes the ferritin light chain, and PIGR (rank 5) which encodes the polymeric Ig receptor. Interestingly, MALAT1, which is a long non-coding RNA, was by far (~2-fold greater than B2M) the most highly expressed HINT transcript.

### HINT Expression Diversity

We sought to identify genes whose expression displayed particularly low or high variability in the HINT. Shown in Fig. 4B, are the 10 genes with the highest and lowest robust CV. Some of the genes with the highest robust CV had identifiable immune- and/or infection-related functions (e.g., IL1B, CXCR1), while others did not. We performed pathway analysis using the 200 genes identified as having the highest variation, and the significant canonical pathways identified are reported in Supplemental Fig. 2. Most prominent among the pathways represented by genes with variability in expression were those related to stress response (EIF2 signaling, Unfolded protein response and Protein ubiquitination) and mitochondrial function (Oxidative phosphorylation and Mitochondrial dysfunction).

Genes with expression mean or variance differences associated with intrinsic (gender, race, etc.) and extrinsic (delivery method, environmental tobacco exposure, etc.) variables were then identified. We detected significant changes in mean expression associated with race, gender, feeding method and delivery method (Table 2). Differences in gene expression variance were also noted for many of these variables (Table 2). For instance, 11 genes demonstrated expression variance associated with environmental tobacco smoke exposure (ETS). Interestingly, all of these 11 genes showed higher variance in subjects exposed to ETS (Fig. 4C).

We also tested nasal turbinates for the presence of viruses or potentially pathogenic bacteria. One or more of these microbes was detected by PCR in 16 of 53 subjects; 11 with *M. catarrhalis,* 1 with *S. pneumoniae,* 1 with *H. Influenzae,* 7 with rhinovirus and 2 with coronavirus. The presence of one of these known pathogens was the variable most profoundly associated with differences in HINT gene expression (Table 2). For instance, many genes displayed significant changes in expression variance associated with the presence of either bacteria (n = 16 genes) or virus (n = 123 genes). Notably, the mean expression of 130 genes was significantly different in subjects with a pathogen when compared to subjects where these pathogens were undetectable. Interestingly, these gene expression changes were more highly associated with the presence of viral pathogens (9 subjects, 282 genes) than bacterial pathogens (12 subjects, 1 gene). Pathway analysis indicated that genes associated with the presence of viral pathogens were involved in tight junction, PI3K/AKT, apoptosis, CD27, lymphotoxin B receptor and GADD45 signaling pathways, as well as DNA double-strand break repair (Supplemental Fig. 3).

**Figure 5:**
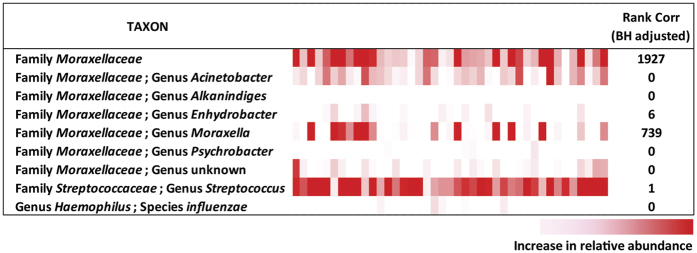
Gene expression associated with microbial burden. The abundance of all taxonomical units for selected pathogens was defined by 16S sequencing and assessed for correlation with host gene expression. Taxonomical units for each of the pathogens are presented as individual rows, while each subject is represented by a column. A relatively high abundance for each taxon is indicated by red. A large number of genes were significantly associated with the burden of any Moraxellaceae family taxon, and specifically for genus *Moraxella*, whereas no appreciable gene expression was associated with the abundance of other pathogens.

These data suggested that nasal gene expression responses to the presence of different pathogenic bacteria (such as *H. influenzae* or *S. pneumoniae*) were more diverse than responses to different common respiratory viruses (such as rhinovirus and coronavirus). To explore this issue further, we comprehensively assessed the relationship between the HINT and the presence of all colonizing bacteria in the nasal turbinates. The abundance of various taxonomical units representing pathogenic species, and their related genera, was quantified by 16S ribosomal sequencing, and correlated with nasal gene expression patterns (Fig. 5). While gene expression patterns were generally not significantly associated with the abundance of *Streptococcus* (1 gene) or *Haemophilus* (0 genes), the expression of 1927 genes was significantly associated with the abundance of any *Moraxellaceae* family. Moreover, the abundance of the pathogenic genus of *Moraxella* was almost solely associated with nasal gene expression (739 genes), while other non-pathogenic genera were almost never associated with nasal gene expression. Pathway analysis indicated that *Moraxella* responsive genes were involved in axonal guidance, A- and B- adrenergic, sphongosine−1-phosphate, IL1, CCR5 and IL17F signaling, retinol biosynthesis, regulation of cytokine production and DNA double-strand break repair (Supplemental Fig. 4).

Finally, in an effort to identify the most robust gene expression patterns, we completed a multivariate analysis using linear regression to control for potential confounding factors. This approach had limited power, given the large number of variables available. Therefore, we chose to include only variables strongly associated with gene expression based upon our marginal (univariate) analyses, including gender and the presence of a known pathogenic virus or *Moraxella* abundance. We also considered two-way interactions among these variables. Following a Benjamini-Hochberg multiple testing procedure to control FDR at a level of 0.05, we identified a total of 49 genes whose expression was significantly associated with any variable (Supplemental Table 1). As might be anticipated, a large proportion of these genes were associated with gender and are located on the sex chromosomes. However, we also identified a number of genes significantly associated with the presence of a known pathogen (rhinovirus, coronavirus or *Moraxella*), in combination with each other, or in combination with gender.

### Gene Expression Validation

**Figure 6:**
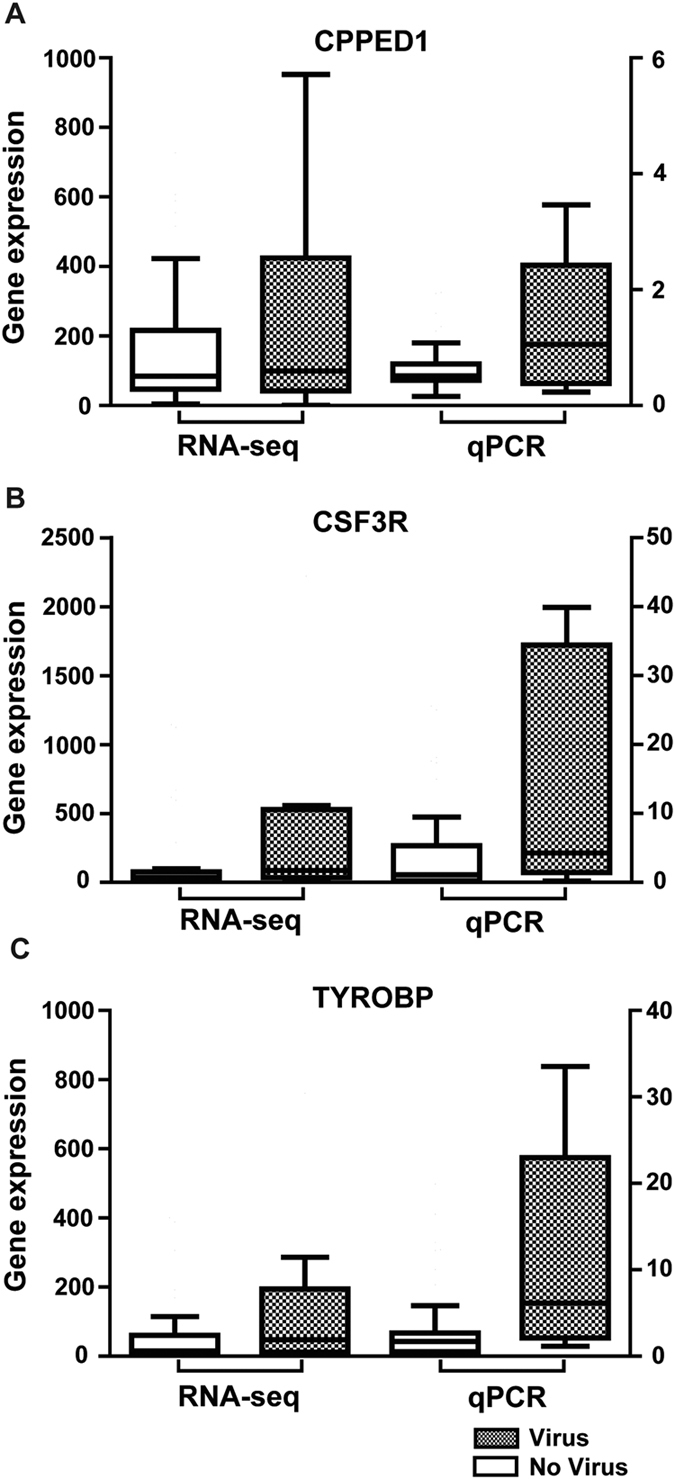
qPCR validation of virus-related expression variance. We performed qPCR to confirm differences in gene expression variance associated with the presence or absence of detectable virus in the nares of healthy infants. Shown are median, upper quartile and lower quartile expression for CPPED1 (panel A), CSF3R (panel B) and TYROBP (panel C). The left side of each panel displays absolute expression estimates based upon RNA-seq. The right side of each panel displays relative expression determined by qPCR. Data from subjects with detectable virus (n = 9) are in grey, while data from subjects without detectable virus (n = 41) are in white.

We used quantitative real-time PCR (qPCR) to validate RNA-seq-based expression estimates for selected genes displaying significant expression changes. We found a very high degree of correlation between RNA-seq- and qPCR-based estimates for LPLUNC (*p *< 7 × 10E-10) and SPLUNC (*p *< 2 × 10E-23). Of 13 additional genes selected for variable-specific expression validation, 11 (85%) demonstrated significant correlation between qPCR and RNA-seq-based estimates. We attempted validation for 5 genes with significant mean differences based upon the presence of any known infant nasal pathogen. Significant differences (*p *< 0.05) in expression were validated for three of these genes including DNAI2, DNAH5 and CES1, while a fourth showed a trend for validation (OAS1, *p *= 0.06; Table 3). We also validated 4 of 7 genes (GPI, KRT8, TYROBP and CES1, p < 0.05) correlated with the abundance of *Moraxella* (Table 4). Finally, we explored 3 genes with expression variation differences associated with the presence of any virus. qPCR data for all three of these genes clearly displayed substantive differences in variance associated with the presence of viral pathogens (Fig. 6).

## Discussion

Responses by resident cells within the respiratory system are likely to play a key role in determining the severity of disease, including illnesses related to infectious pathogens. Others have used an assessment of the nasal transcriptome to assess disease-associated changes in airway gene expression in older pediatric or adult subjects^4^,^5^. Although it is highly challenging to sample the lung or lower airways in young subjects, our data demonstrate successful identification of the healthy infant nasal transcriptome (HINT). We also found a high degree of similarity with published results of the nasal and intra-pulmonary airways. Particularly, we found a very high proportion of HINT non-housekeeping genes are also expressed in the upper and lower airways of adults^9^,^10^. Thus, we suggest that this approach is useful to estimate responses that occur in the lower airways of infants.

Our data are consistent with a recent report of the nasal transcriptome in an older population^4^. A direct comparison of our HINT with gene expression from control subjects from that study demonstrated a high degree of similarity of the nasal transcriptome irrespective of age. It should be noted that this comparison of similarity does not take into account variation in expression levels across subjects and airway regions. Clearly, significant differences in gene expression exist between nasal and lower airway epithelial cells. However, our data do support the study of the nasal transcriptome in infants and children as a reasonable surrogate for assessing changes in airway gene expression during diseased states.

We directly assessed variation in detected nasal expression patterns from healthy infants and found sets of genes with either high or low variance. Genes with low variance (EIF5, CANX, TAF7, SEPT7) may represent those under highest selective pressure and may represent targets of key regulatory factors. Genes with high variance (PROK2, IL1B, CXCR1) could implicate factors that may be associated with heterogeneity in resilience versus susceptibility to disease. Interestingly, many genes with inherently high variance were associated with immunity and infection responses, which may be associated with changes in cellular composition. Differences in expression variance were also identified in association with many demographic and host variables, particularly the presence of known respiratory pathogens in these asymptomatic infants. By comparison, most demographic variables were associated with modest differences in mean expression levels.

Comparing gene expression changes in subjects with detectable virus or potentially pathogenic bacteria in the nares, we found greater effect sizes associated with the presence of rhinovirus or coronavirus when compared to any pathogen or bacterial pathogens alone. We suspected these data were likely due to greater variability in gene expression associated with distinct bacteria. Using 16S microbial sequencing of nasal samples from these subjects, we found a strong association between gene expression and the presence of pathogenic *Moraxella* genera, but not with non-pathogenic *Moraxella* or other pathogenic bacteria. Furthermore, multivariate analyses identified gene expression patterns significantly associated with the interaction of pathogen and gender. Our results suggest that the presence of bacterial or virus are strongly associated with nasal gene expression, even in asymptomatic infants, but that responses are not identical in males and females.

We recognize some limitations of the current study. Although we were able to recover RNA from a vast majority of the subjects using our detailed protocol (see Supplement), we saw significant variability in yield across subjects. Therefore, we chose to use a commercially available “low input” protocol for RNA sequencing, which enabled capture of data from all subjects. Although it is likely that this approach sacrifices sensitivity for detection of genes expressed at a low level, we did observe very high technical replicate correlation. We also noted sample-wide correlations which were consistent, but somewhat lower than expected (r = 0.730 ± 0.148). We believe this reflects the heterogeneity of individual samples, which are likely representative of multiple (epithelial, as well as some non-epithelial) cell types. Unfortunately, in the current study, we were unable to use cytometry to directly assess the diversity of cells within individual subjects, or among subjects across the cohort. However, sample composition was partially assessed using cell-specific marker gene expression. High levels of nasal-specific and general epithelial cell marker expression were identified. These data also suggested a low level (but not absence) of immune/inflammatory cells, which may be greater without washing of the nares prior to cell recovery. In addition, the consistently absent or low detection of globin transcripts indicated no significant injury from the procedure as well as little or no red blood cell confounding of expression data. These results strongly suggest that this method predominantly assesses nasal epithelial cell gene expression. It is also worth noting that we found differences in sample recovery efficiency based upon the swab used for brushing the nasal turbinates. After some trial and error, and consideration of the relationship between aggressive cell recovery and injury to the subjects, we were successful in consistently recovering sufficient RNA for complete transcriptome analysis and expression validation. Alternate brush choices, allowing more aggressive brushing, may be better for older subjects. Finally, our recovery procedures were affected by apparent changes in buoyancy of cells stored in RNA stabilizer. In order to overcome some observed variability in centrifugation-based cell recovery of cells suspended in RNA stabilizer, we ensured 100% recovery by simple centrifugation through a membrane. To overcome potential technical impediments, we strongly recommend following the specific protocol provided, particularly the choice of swab.

Regardless of the limitations, the data reported here demonstrate both the fidelity and feasibility of our methodology. These data also serve as a benchmark for healthy infant airway gene expression, describing normal expression and variation within a healthy infant cohort. Future application of these procedures may facilitate the identification of gene expression patterns that distinguish diseased and normal states. These methods could also provide novel information about potential disease mechanisms and efficacy of therapeutic interventions.

## Methods

For complete methods, please refer to Supplemental material.

### Cohort Recruitment and Sampling

All procedures were reviewed and approved by the University of Rochester Research Subjects Review Board and the Rochester General Hospital Clinical Investigation Committee, and approved by the NIAID Division of Microbiology and Infectious Disease. All studies were carried out in accordance with these approved guidelines. Written informed consent was obtained from all subjects (and their families).

Healthy, full term newborns (≥37 weeks gestation) were recruited at approximately one month. Nasal samples were obtained after washing, essentially as previously described^5^,^11^. For RNA recovery, the swab was immediately placed in RNA stabilizer (RNAprotect, Qiagen, Germantown, MD) and stored at 4 °C. Cells were recovered by filtering through a 0.45 uM membrane filter. Cells were lysed, homogenized by passing through a 28 g needle and total RNA was recovered (AbsolutelyRNA Miniprep kit, Agilent, Santa Clara, CA), according to manufacturer’s instructions.

### Library Preparation and Sequencing

Approximately 1ng of total RNA was amplified using the SMARter Ultra Low amplification kit (Clontech, Mountain View, CA). Libraries were constructed using the NexteraXT library kit (Illumina, San Diego, CA). Libraries were sequenced on the Illumina HiSeq2500 to generate ~20 million 1 × 100-bp single end reads per sample.

### Read Mapping, Data Normalization and Filtering

Sequences were aligned against human genome version of hg19 using TopHat^12^, counted with HTSeq^13^ and normalized for total counts (reads per million, RPM). We used a non-specific filtering strategy to remove genes with low expression values indistinguishable from background signal. These data area available in dbGaP; accession number phs001201.v1.p1.

### Gene Significance Analyses

We calculated a robust measure of expression variance for each gene, based upon the relationship between inter-quartile range and median values. For categorical variables such as gender and delivery method, we used SAMseq to identify genes with significant differences in mean expression (FDR < 0.05). For continuous variables such as gestational age and microbiome derived variables, we used both Pearson and Spearman correlation tests, with the Benjamini-Hochberg correction^14^, to select significant genes (adjusted *p*-value < 0.05). To better understand the association between gene expression and clinical/demographic variables, we also used Levene’s test for equal variance^15^, with the Benjamini-Hochberg correction, to select genes that have significantly different variances between groups (adjusted *p*-value < 0.05). We performed canonical pathway analyses using Ingenuity Pathway Analysis software (Qiagen) to identify significant pathways (*p*-value < 0.05).

### Multivariate Regression Analyses

We conducted a multivariate regression analysis between gene expression and sex, race, gestational age, birth weight, environmental smoke exposure, birth delivery method, breast feeding, and asymptomatic presence of virus or bacterial pathogen. Multiple linear regression, based on least square fitting criteria, was used to analyze the linear association between clinical/demographic factors (covariates) and gene expression levels (response variable). Goodness-of-fit *F*-test was applied to test the significance of overall linear association, with FDR controlled at 0.05 level by the Benjamini-Hochberg procedure. If a gene was selected as significant, we performed regression *t*-test to identify significant covariates (*p*-value < 0.05) for this gene.

### Detection of Pathogenic Virus and Bacteria

TaqMan Array Card (TAC) technology was used to detect for the presence of respiratory viral pathogens and several potentially pathogenic bacteria in the nasal washings of subjects, as described^16^,^17^. The TAC detects respiratory syncytial virus (RSV), rhinovirus, coronavirus (1–4), *Haemophilus influenzae* and *Streptococcus pneumoniae,* among others. In addition, the presence of *Moraxella catarrhalis* was assessed by real-time PCR using published primers and probes, essentially as previously described^18^,^19^.

### Microbiome Analysis

Matched nasal swab specimens were collected. V3-V4 16S rRNA was amplified from total genomic DNA and sequenced on an Illumina MiSeq (Illumina, San Diego, CA). 16S rRNA bacterial sequence reads were assessed for quality and analyzed using phylogenetic and Operational Taxonomic Unit (OTU) methods in the Quantitative Insights into Microbial Ecology (QIIME) software, version 1.9 20. For the purposes of OTU relative abundance analysis, the raw OTU table was normalized using the cumulative sum stabilization method from the metagenomicSeq R package^21^.

### Gene Expression Validation

Quantitative real-time polymerase chain reaction (qPCR) was performed using gene-specific primer sets and Taqman chemistry on a ViiA 7 Real-Time PCR System (Life Technologies). Gene expression levels were calculated relative to *PPIA* (cyclophilin A) using the ddCT method.

## Additional Information

**How to cite this article**: Chu, C.-Y. *et al*. The Healthy Infant Nasal Transcriptome: A Benchmark Study. *Sci. Rep.* **6**, 33994; doi: 10.1038/srep33994 (2016).

## Supplementary Material

Supplementary Information

## Figures and Tables

**Table 1:** Characteristics of the Infant Cohort.

Demographic Variables	Healthy Infants (n = 53)
Age, month	1.04 ± 0.16
Gestational age, week	39.34 ± 1.04
Birth weight, kg	3.3 ± 0.4
Gender, female/male	29/24
Race, Caucasian/African American	37/10
Delivery method, vaginal/caesarian section	37/16
Breastfed, yes/no	40/13
Tobacco smoke exposure, yes/no	10/43
Family size	3.17 ± 1.35
Any pathogen detected, yes/no	16/34
Virus detected, yes/no	9/41
Bacteria detected, yes/no	12/38

**Table 2:** Genes displaying mean expression differences or expression variance differences associated with demographic and subject variables.

Variable	Mean Differences (#genes)	Variance Differences (#genes)
Gender, 29 F vs. 24 M	16	29
Delivery method, 16 caesarian vs. 37 vaginal	7	25
Breastfed, 40 yes vs. 13 no	19	15
Tobacco smoke exposure, 10 yes vs. 43 no	0	11
Race, 37 Caucasian vs. 10 African American	76	5
Pathogen detected, 16 positive vs. 34 negative	130	25
Virus detected, 9 positive vs. 41 negative	282	123
Bacteria detected, 12 positive vs. 38 negative	1	16

Shown is the number of genes with expression differences associated with each variable.

**Table 3:** qPCR was used to validate the expression of genes displaying differences in expression between subjects with detectable virus (n = 9) and those without (n = 41).

Gene	Fold change of qPCR	Fold change of RNA-seq	MWU
DNAI2	0.45	0.27	0.0104
DNAH5	0.42	0.31	0.0276
OAS1	2.23	1.89	0.0618
B2M	0.63	1.68	0.0927
CES1	0.04	0.20	1.47E-07

Shown are fold-change estimates based upon RNA-seq absolute expression estimates and qPCR relative expression estimates. Shown also are non-parametric p-values for tests of significant differences based upon qPCR data.

**Table 4:** qPCR was used to validate genes displaying expression patterns associated with the abundance of genus *Moraxella*.

Gene	Correlation	p-value
CD83	−0.1080	0.6905
FOXRED1	−0.2885	0.2971
GPI	−0.5547	0.0208
KRT8	−0.5186	0.0329
SOCS6	−0.3991	0.1257
TYROBP	−0.4862	0.0023
CES1	−0.5987	0.0008

Shown are rank correlation coefficients (r), comparing gene expression determined by qPCR and genus *Moraxella* abundance determined by 16S sequencing. Also shown are p-values associated with these correlation coefficients.
