# Supplementary material for: The Healthy Infant Nasal Transcriptome: A Benchmark Study
Source: Sci Rep. 2016 Sep 23;6:33994. doi: 10.1038/srep33994 (PMC5034274; doi:10.1038/srep33994)

Online Data Supplement for:

The Healthy Infant Nasal Transcriptome: A Benchmark Study

*Chin-Yi Chu[1], *Xing Qiu[3], Lu Wang[3], Soumyaroop Bhattacharya[1], Gerry Lofthus[5], Anthony Corbett[3], Jeanne Holden-Wiltse[3], Alex Grier[4], Brenda Tesini[2], Steven R. Gill[4], Ann R. Falsey[6], Mary T. Caserta[2], Edward E. Walsh[6] and Thomas J Mariani[1,5]

Supplemental Tables: 2
Supplemental Figures: 4

Supplemental Methods

Cohort Recruitment and Sampling

All procedures were reviewed and approved by the University of Rochester Research Subjects Review Board and the Rochester General Hospital Clinical Investigation Committee, and approved by the NIAID Division of Microbiology and Infectious Disease. Healthy, full term newborns (≥37 weeks gestation) were recruited into this study during their birth hospitalization from two hospitals in Rochester, NY as part of a larger study of respiratory syncytial virus (RSV) infection severity of the Respiratory Pathogen Research Center at the University of Rochester. Enrollment occurred during the late summer and fall of 2012-2013 and 2013-2014. At one month of age subjects were seen by the study team, and three nasal samples (a nasal swab, a nasal wash and a nasal brushing specimen) were obtained if they were well and free of respiratory symptoms. If a subject was ill at one month of age the visit was postponed for approximately two weeks until the infant was again asymptomatic. The procedure of collecting nasal epithelial cell for transcriptomic analysis was modified previous published method[1]. Prior to brushing, the nasal cavity was washed with sterile non-bacteriostatic saline to remove any mucus, debris or intra-airway inflammatory cells, as previously described[2]. Immediately following, a nasal brush sample was obtained from the same nares using a sterile Copan flocked swab (FLOQSwabs, product #518C, Copan Diagnostics, Murrieta CA) that was inserted completely into the anterior nares and rotated with an up and down circular motion for approximately 3-5 seconds to recover cells from the inferior turbinate. The swab was immediately placed in RNA stabilizer (RNAprotect, Qiagen, Germantown, MD) and stored at 4°C. All subjects tolerated the procedures without difficulty. A second nasal swab was also collected from the contralateral nostril without washing for microbiomics analysis.

Sample Collection and Processing Protocol for Nasal Brush Specimens

1. Place a dry cloth towel on the infant's chest under the chin.
2. Pour 4-5 ml of sterile phosphate buffered saline into a sterile cup and aspirate the saline into a sterile rubber irrigation bulb.
3. With the infant's head at a greater than 45 degree upward angle, the bulb is placed into the nostril until it occludes the nostril.
4. Quickly compress and decompress the bulb to force the fluid into and back out of the nostril rapidly.
5. Observe the infant for several minutes to assess for difficulty breathing.
6. Place a flocked swab (FLOQSwabs, Copan Diagnostics, Murrieta CA) gently into the nares that was just washed and rotate to cover all surfaces. Move the flocked swab up and down slightly while brushing the mucosa for 5 seconds.
7. Remove the swab and place it into RNAprotect solution (2 ml).
8. Cap tube and vortex vigorously for 10 sec, to ensure that all cells come into contact with RNAprotect solution.
9. Observe the subject for 5 minutes to assure there is no bleeding.
10. Samples should be processed into lysis buffer immediately (same day), but can be stored at 4 degrees C at this point for up to 1 week. Before processing, vortex vigorously for 10-20 seconds and remove swabs from tube, taking care to retain as much RNAprotect solution as possible.
11. Place 600 ul of RNAprotect solution over a .45 um filter cup and spin at 14000 rpm for 60 seconds. Pour off filtered RNAprotect solution and repeat this process until all RNAprotect

has been filtered. If the filter clogs, transfer any solution left on top of the filter to a new filter cup and continue.

12. Add 250 ul RNA lysis buffer containing BME directly to the filter and lyse by passing solution repeatedly (X10-12) through new unused insulin needle (0.5cc, 28g) with syringe. Leave the lysed solution on the filter and spin at 14000 rpm for 60 seconds. If more than one filter was used, transfer the lysed solution to the next filter and repeat. If necessary, lysed and homogenized cells can be stored at -20C or below. If using frozen lysates, thaw for 20 minutes on ice and proceed with step 9.
13. Add 250 µl of 70% ethanol to the tube with the lysate and vortex the tube for 5 seconds or until mixed thoroughly.
14. Transfer the ~500 µl of this mixture to an RNA Binding Spin Cup that is seated in a 2-ml receptacle tube and cap the spin cup. Vigorously flex the hinge of the spin cup, prior to closing it, so that it becomes flexible and the cap can be firmly seated in the tube, to reduce the chance of leakage during vortexing.
15. Spin in a microcentrifuge at 14000 rpm for 60 seconds.
16. Remove and retain the spin cup and transfer the filtrate to another tube and store it at -20°C or below. Replace the spin cup in the receptacle tube.
17. Add 600 µl of 1× Low-Salt Wash Buffer and snap the cap of the receptacle tube onto the spin cup. Spin in a microcentrifuge at 14000 rpm for 60 seconds.
18. Remove and retain the spin cup and discard the filtrate (to hazardous waste). Replace the spin cup in the receptacle tube and cap the spin cup. Spin the tube in a microcentrifuge at 14000 rpm for 2 minutes.

19. Prepare the DNase solution by gently mixing 50 µl of DNase Digestion Buffer with 5 µl of reconstituted RNase-Free DNase I.
20. Add the DNase solution directly onto the matrix inside the spin cup and cap the spin cup.
21. Incubate the sample at 37°C for 30 minutes in an air incubator/oven.
22. Add 600 µl of 1× High-Salt Wash Buffer to the spin cup and cap the spin cup. Spin in a microcentrifuge at 14000 rpm for 60 seconds.
23. Remove and retain the spin cup and discard the filtrate. Replace the spin cup in the receptacle tube.
24. Add 600 µl of 1× Low-Salt Wash Buffer and cap the spin cup. Spin in a microcentrifuge at 14000 rpm for 60 seconds.
25. Remove and retain the spin cup and discard the filtrate. Replace the spin cup in the receptacle tube.
26. Add 300 µl of 1× Low-Salt Wash Buffer and cap the spin cup. Spin in a microcentrifuge at 14000 rpm for 2 minutes to dry the matrix.
27. Transfer the spin cup to a new 1.5-ml microcentrifuge (collection) tube and discard the 2-ml receptacle tube.
28. Warm the Elution Buffer at 60°C for at least 5 minutes.
29. Add 30 µl of Elution Buffer directly onto the center of the matrix inside the spin cup and cap the spin cup. Incubate the tube for 5 minutes at room temperature. Spin in a microcentrifuge at 14000 rpm for 60 seconds.
30. Repeat the elution step by pipetting the elution buffer from the collection tube back onto the matrix (using the same buffer from step 64). Incubate for 5 minutes at room temperature. Spin in a microcentrifuge at 14000 rpm for 60 seconds.

RNA Recovery

Nasal epithelia cells were recovered from stabilizer solution following vigorous vortexing by filtering through a 0.45 uM membrane filter. Cells were lysed immediately following filtration by the addition of RNA lysis buffer (AbsolutelyRNA Miniprep kit, Agilent, Santa Clara, CA) to the filter membrane. The lysates were homogenized by passing through a 28g needle and total RNA was recovered from lysates using solid-phase affinity, according to manufacturer's instructions.

Library Preparation and Sequencing

Approximately 1ng of total RNA was amplified using the SMARter Ultra Low amplification kit (Clontech, Mountain View, CA) with PCR amplification to generate enough yield for library construction. Amplified cDNA quantification was determined with the Qubit Flourometer (Life Technologies, Grand Island, NY) and quality was assessed using the Agilent Bioanalyzer 2100 (Santa Clara, CA). Libraries were constructed using the NexteraXT library kit (Illumina, San Diego, CA) per manufacturer's recommendations. Nextera libraries were quantified with the Qubit Flourometer (Life Technologies) and quality was assessed using the Agilent Tape Station. Libraries were sequenced on the Illumina HiSeq2500 to generate ~20 million 1x100-bp single end reads per sample.

Read Mapping, Data Normalization and Filtering

Sequences were aligned against human genome version of hg19 using TopHat[3]. Raw counts were generated with HTSeq[4] and normalized for total counts by dividing the raw count for each individual gene in a particular sample by the sum total of counts for all the genes in that sample

(reads per million, RPM). We used a non-specific filtering strategy to remove genes with low expression values indistinguishable from background signal. We first computed M_i, the 95% quantile for the *i*th gene expression among all subjects, for all genes. Next, we defined trimmed maximum log-expressions as $L_i = \log_2(M_i + 1)$ and pooled all L_i s to form a density plot (Supplemental Figure 1). Two clusters of gene expression were identified; those with low expression (L_i < 3.0 or M_i <7.0) and those with higher expression ($L_i \geq$ 3.0 or $M_\mathrm{i} \geq$7.0). Genes with low expression were removed, resulting in an analytical data set containing 13,978 genes.

Gene Significance Analyses

We measured the variation of each gene by $r_i = \frac{\mathrm{IQR}_i}{\mathrm{Med}_i}$, where IQR_i is the inter-quartile range of this gene and Med_i is the sample median. r_i can be considered as the robust coefficient of variation of the *i*th gene. Genes with the highest and lowest variation ranked by r_i were identified. For categorical variables such as gender and delivery method, we used SAMseq[5] to identify significant genes, with false discovery rate (FDR) controlled at the 0.05 level (q-value<0.05). For continuous variables such as gestational age and microbiome derived variables, we used both Pearson and Spearman correlation tests, with the Benjamini-Hochberg correction[6], to select significant genes (adjusted p-value<0.05). To better understand the association between gene expression and clinical/demographic variables, we also used Levene’s test for equal variance[7], with the Benjamini-Hochberg correction, to select genes that have significantly different variances between groups (adjusted p-value<0.05). We performed canonical pathway analyses using Ingenuity Pathway Analysis software (Qiagen). Each identifier was mapped to its

corresponding object in Ingenuity's Knowledge Base. A pathway with a p-value<0.05 was classified significant.

Multivariate Regression Analyses

We conducted a multivariate regression analysis between gene expression and the following variables: sex, race (with three separate binary variables, Caucasian, African, and Asian), environmental smoke exposure, birth delivery method, breast feeding, and asymptomatic presence of pathogen (technical details can be found in the next section). Gestational age and birth weight were also included to control for their possible confounding effects. Multiple linear regression, based on least square fitting criteria, was used to analyze the linear association between clinical/demographic factors (covariates) and gene expression levels (response variable). Goodness-of-fit F-test was applied to test the significance of overall linear association, with FDR controlled at 0.05 level by the Benjamini-Hochberg procedure. If a gene was selected as significant, we performed regression t-test to identify significant covariates (p-value <0.05) for this gene.

Detection of Viruses and Pathogenic Bacteria

A separate flocked swab (Copan, FLOQSwabs™ catalog # 525CS01, Copan, Murrieta, CA) was used to obtain a sample from the opposite nares and immediately placed into 2 ml of sterile PBS and shaken prior to being placed on ice and transported to the laboratory. Total nucleic acid was extracted using 200 µL of sample with the QIAamp® Viral RNA Mini Kit on a QIAcube (Qiagen, Valencia, CA) with a final elution volume of 75 µL. Recombinant RNasin® Ribonuclease Inhibitor (2 µL, Promega, Madison, WI) was added to each nucleic acid sample.

TaqMan® Array Card (TAC) technology was used on the ViiA7 instrument (Life Technologies, Carlsbad, CA) to perform real time PCR (rPCR) in a non-multiplexed format. The 384 well micro-fluidic card (Life Technologies, Carlsbad, CA) was preloaded with primer and probe sets to detect 23 viral and bacterial pathogens plus internal controls in duplicate as previously described[8,9], with primer and probe modifications as outlined in Supplemental Table 2. Viral targets included influenza A and B, respiratory syncytial virus (RSV), parainfluenza virus (PIV) 1, 2 and 3, rhinovirus, enterovirus, adenovirus, coronavirus 1 through 4, human metapneumovirus (hMPV), bocavirus (hBoV), and parechovirus. The card also detects *H. influenzae*, *S. pneumoniae*, *M. pneumoniae*, *C pneumoniae*, *M. hominis*, Ureaplasma, and *B. pertussis*.

Seven samples and one no-template control were assayed per card using 20 µL of sample with AgPath-ID™ One-Step RT-PCR Reagents (Applied Biosystems, Foster City, CA). The thermocycling conditions were 45°C for 10 minutes, 94°C for 10 minutes, and 45 cycles of 94°C for 30 seconds followed by 60°C for 1 minute. A sample was considered positive for a pathogen when the duplicate wells registered a rPCR curve crossing the threshold. Discrepant results were confirmed in individual rPCR assays performed in a 96 well format on a Life Technologies 7500 real time PCR system (Life Technologies, Carlsbad, CA).

The presence of *Moraxella cataarrhalis* was assessed separately by real-time PCR essentially as described, with minor modifications[5,10]. Primer and probe sequences were similar to these prior studies (300 nM; 5'-GTGAGTGCCGCTTTACAACC-3', 5'-TGTATCGCCTGCCAAGACAA-3'), with the exception that the probe was labeled with FAM (200nM; 5'FAM-TGCTTTTGCAGCTGTTAGCCAGCCTAA-BHQ1). Reactions were performed on an ABI 7500 instrument in a 25 ul reaction mixture, which consisted of 5 ul of extracted nucleic acid,

300 nM of each primer, 200 nM of probe and AgPath-ID One-Step RT-PCR Reagents (Life Technologies). The reaction included one cycle at 45°C for 10 minutes, followed by one cycle at 95°C for 10 minutes, and 45 cycles of 95°C for 15 seconds and 60°C for 1 minute.

Microbiome DNA Extraction and Sequencing

Matched nasal specimens for microbiome analysis were collected from the anterior nares of the opposing nasal cavity. After collection, the flocked swab was placed into sterile UV-treated PBS, immediately placed on ice and stored at 4° C prior to processing and storing at -80° C. Total genomic DNA was extracted from brushings of contralateral nares used for RNA collection as previously described[11-13]. V3-V4 16S rRNA was amplified with Phusion High-Fidelity polymerase (Thermo Scientific, Waltham, MA) using dual-indexed coded primers, normalized, pooled and paired-end sequenced (2 X 300bp) on an Illumina MiSeq (Illumina, San Diego, CA) in the University of Rochester Genomics Research Center[14]. Each sequencing run included: (1) positive controls consisting of a 1:5 mixture of *Staphylococcus aureus*, *Lactococcus lactis*, *Porphyromonas gingivalis*, *Streptococcus mutans*, and *Escherichia coli*; and (2) negative controls consisting of sterile saline.

16S rRNA Sequence Processing

The 16S rRNA bacterial sequence reads were assessed for quality and analyzed using phylogenetic and Operational Taxonomic Unit (OTU) methods in the Quantitative Insights into Microbial Ecology (QIIME) software, version 1.9[15]. Read pairs were assembled using fastq-join from the ea-utils package[16], requiring at least 40 bases of overlap and allowing a maximum of 10% mismatched bases. Operational taxonomic units (OTU) were picked using the reference-

based USEARCH (version 5.2)[17] pipeline in QIIME, using the May 2013 release of the GreenGenes 99% OTU database[18]. Chimera detection and removal was performed *de novo* using UCHIME[19]. OTU clusters with less than four sequences were removed, and representative sequences used to make taxonomic assignments for each cluster were selected on the basis of abundance. The RDP Naïve Bayesian Classifier was used for taxonomic classification with the GreenGenes reference database, using a minimum confidence threshold of .85 and otherwise default parameters[20].

OTU Table Analysis

For the purposes of α-diversity calculations, twenty iterations of rarefaction at an even depth of 3,900 reads per sample were performed. The mean α-diversity over all rarefactions was computed for each sample using QIIME's PD Whole Tree metric, the Chao 1 index[21], and the raw count of OTUs observed. For the purposes of OTU relative abundance analysis, the raw OTU table was normalized using the cumulative sum stabilization method from the metagenomicSeq R package[22]. The normalized table was filtered to contain only taxa phylogenetically proximate to three known potential pathogens of interest. These were OTUs corresponding to: the species *Haemophilus influenzae, Moraxella catarrhalis*, and *Streptococcus pneumonia*, where available; all individual genera contained within the same three families as these species; and the three families themselves.

Gene Expression Validation

Quantitative real-time polymerase chain reaction (qPCR) was performed using aliquots of RNA samples obtained for sequencing. cDNA was generated from approximately 250 ng RNA

using the iScript cDNA Synthesis Kit (BioRad, Hercules, CA) according to manufacturer's recommendations. PCR was performed using gene-specific primer sets (http://pga.mgh.harvard.edu/primerbank/) and Taqman chemistry (Universal Master Mix II with UNG, Life Technologies) on a ViiA 7 Real-Time PCR System (Life Technologies). Gene expression levels were calculated relative to *PPIA* (cyclophilin A) using the ddCT method.

References

1 Zhang, X. *et al.* Similarities and differences between smoking-related gene expression in nasal and bronchial epithelium. *Physiological genomics* **41**, 1-8 (2010).
2 Hall, C. B. & Douglas, R. G., Jr. Clinically useful method for the isolation of respiratory syncytial virus. *The Journal of infectious diseases* **131**, 1-5 (1975).
3 Kim, D. *et al.* TopHat2: accurate alignment of transcriptomes in the presence of insertions, deletions and gene fusions. *Genome biology* **14**, R36 (2013).
4 Anders, S., Pyl, P. T. & Huber, W. HTSeq--a Python framework to work with high-throughput sequencing data. *Bioinformatics* **31**, 166-169 (2015).
5 Desai, H. *et al.* Bacterial colonization increases daily symptoms in patients with chronic obstructive pulmonary disease. *Annals of the American Thoracic Society* **11**, 303-309 (2014).
6 Benjamini, Y. & Hochberg, Y. Controlling the false discovery rate: a practical and powerful approach to multiple testing. *Journal of the Royal Statistical Society. Series B (Methodological)*, 289-300 (1995).
7 Levene, H. Robust tests for equality of variances1. *Contributions to probability and statistics: Essays in honor of Harold Hotelling* **2**, 278-292 (1960).
8 Harvey, J. J. *et al.* Comparative analytical evaluation of the respiratory TaqMan Array Card with real-time PCR and commercial multi-pathogen assays. *Journal of Virological Methods* **228**, 151-157 (2016).
9 Kodani, M. *et al.* Application of TaqMan low-density arrays for simultaneous detection of multiple respiratory pathogens. *Journal of clinical microbiology* **49**, 2175-2182 (2011).
10 Greiner, O., Day, P. J., Altwegg, M. & Nadal, D. Quantitative detection of Moraxella catarrhalis in nasopharyngeal secretions by real-time PCR. *Journal of clinical microbiology* **41**, 1386-1390 (2003).
11 Dardas, M. *et al.* The impact of postnatal antibiotics on the preterm intestinal microbiome. *Pediatric research* **76**, 150-158 (2014).
12 Merkley, M. A. *et al.* The effect of antibiotics on the microbiome in acute exacerbations of chronic rhinosinusitis. *International forum of allergy & rhinology* **5**, 884-893 (2015).
13 Zhu, L. *et al.* Characterization of gut microbiomes in nonalcoholic steatohepatitis (NASH) patients: a connection between endogenous alcohol and NASH. *Hepatology* **57**, 601-609 (2013).
14 Fadrosh, D. W. *et al.* An improved dual-indexing approach for multiplexed 16S rRNA gene sequencing on the Illumina MiSeq platform. *Microbiome* **2**, 6 (2014).
15 Caporaso, J. G. *et al.* QIIME allows analysis of high-throughput community sequencing data. *Nature methods* **7**, 335-336 (2010).
16 Aronesty, E. Command-line tools for processing biological sequencing data. (2011).
17 Edgar, R. C. Search and clustering orders of magnitude faster than BLAST. *Bioinformatics* **26**, 2460-2461 (2010).
18 DeSantis, T. Z. *et al.* Greengenes, a chimera-checked 16S rRNA gene database and workbench compatible with ARB. *Applied and environmental microbiology* **72**, 5069-5072 (2006).
19 Edgar, R. C., Haas, B. J., Clemente, J. C., Quince, C. & Knight, R. UCHIME improves sensitivity and speed of chimera detection. *Bioinformatics* **27**, 2194-2200 (2011).

20 Wang, Q., Garrity, G. M., Tiedje, J. M. & Cole, J. R. Naive Bayesian classifier for rapid assignment of rRNA sequences into the new bacterial taxonomy. *Applied and environmental microbiology* **73**, 5261-5267 (2007).

21 Chao, A. Nonparametric Estimation of the Number of Classes in a Population. *Scandinavian Journal of Statistics* **11**, 265-270 (1984).

22 Paulson, J. N., Stine, O. C., Bravo, H. C. & Pop, M. Differential abundance analysis for microbial marker-gene surveys. *Nature methods* **10**, 1200-1202 (2013).

23 CDC. WHO: CDC protocol of realtime RTPCR for swine influenza A (H1N1). 8 (2009).

24 BiosearchTechnologies. *Influenza A Subtyping ValuPanel Reagents licensed from the CDC*, https://www.biosearchtech.com/products/qpcr-and-snp-genotyping/valupanel-reagents/influenza-a-subtyping

25 Fry, A. M. *et al.* The burden of hospitalized lower respiratory tract infection due to respiratory syncytial virus in rural Thailand. *PLoS One* **5**, e15098 (2010).

26 Weinberg, G. A. *et al.* Field evaluation of TaqMan Array Card (TAC) for the simultaneous detection of multiple respiratory viruses in children with acute respiratory infection. *J Clin Virol* **57**, 254-260 (2013).

27 Harvey, J. J. *et al.* Comparative analytical evaluation of the respiratory TaqMan Array Card with real-time PCR and commercial multi-pathogen assays. *J Virol Methods* **228**, 151-157 (2016).

28 Heim, A., Ebnet, C., Harste, G. & Pring-Akerblom, P. Rapid and quantitative detection of human adenovirus DNA by real-time PCR. *J Med Virol* **70**, 228-239 (2003).

29 Dare, R. K. *et al.* Human coronavirus infections in rural Thailand: a comprehensive study using real-time reverse-transcription polymerase chain reaction assays. *J Infect Dis* **196**, 1321-1328 (2007).

30 Lu, X. *et al.* Real-time PCR assays for detection of bocavirus in human specimens. *J Clin Microbiol* **44**, 3231-3235 (2006).

31 Nix, W. A. *et al.* Detection of all known parechoviruses by real-time PCR. *J Clin Microbiol* **46**, 2519-2524 (2008).

32 Meyler, K. L., Meehan, M., Bennett, D., Cunney, R. & Cafferkey, M. Development of a diagnostic real-time polymerase chain reaction assay for the detection of invasive Haemophilus influenzae in clinical samples. *Diagn Microbiol Infect Dis* **74**, 356-362 (2012).

33 Carvalho Mda, G. *et al.* Evaluation and improvement of real-time PCR assays targeting lytA, ply, and psaA genes for detection of pneumococcal DNA. *J Clin Microbiol* **45**, 2460-2466 (2007).

34 Winchell, J. M., Thurman, K. A., Mitchell, S. L., Thacker, W. L. & Fields, B. S. Evaluation of three real-time PCR assays for detection of Mycoplasma pneumoniae in an outbreak investigation. *J Clin Microbiol* **46**, 3116-3118 (2008).

35 Mitchell, S. L., Budhiraja, S., Thurman, K. A., Lanier Thacker, W. & Winchell, J. M. Evaluation of two real-time PCR chemistries for the detection of Chlamydophila pneumoniae in clinical specimens. *Mol Cell Probes* **23**, 309-311 (2009).

36 Ferandon, C. *et al.* Development of a real-time PCR targeting the yidC gene for the detection of Mycoplasma hominis and comparison with quantitative culture. *Clin Microbiol Infect* **17**, 155-159 (2011).

37 Xiao, L. *et al.* Detection and characterization of human Ureaplasma species and serovars by real-time PCR. *J Clin Microbiol* **48**, 2715-2723 (2010).

38 Yi, J., Yoon, B. H. & Kim, E. C. Detection and biovar discrimination of Ureaplasma urealyticum by real-time PCR. *Mol Cell Probes* **19**, 255-260 (2005).

39 Tatti, K. M. *et al.* Development and evaluation of dual-target real-time polymerase chain reaction assays to detect Bordetella spp. *Diagn Microbiol Infect Dis* **61**, 264-272 (2008).

Supplemental Table

Supplemental Table 1. Benjamini-Hochberg adjusted p-values for association between individual genes and variables. Highlighted cells indicate genes demonstrating significant association for individual variables.

Gene	Overall	Gender	Moraxella	Virus	Gender* Moraxella	Gender* Virus	Moraxella* Virus
ACBD3	0.0068	0.9100	0.1120	0.6229	0.6541	0.1107	**0.0005**
B2M	0.0377	0.6730	0.8970	0.9376	0.2811	0.1537	**0.0490**
BEST1	0.0270	0.5690	0.8970	0.1178	0.1486	0.0952	0.1353
CHI3L2	0.0285	0.7670	0.8970	0.3187	0.4481	0.0943	**0.0477**
CLDN25	0.0009	0.5690	**0.0000**	**0.0226**	**0.0001**	**0.0148**	0.0931
CSNK1A1L	0.0045	0.5530	0.2320	0.3942	**0.0302**	0.2843	**0.0289**
DDX3Y	3.2E-08	**4.8E-12**	0.8970	0.6229	0.6160	0.7826	0.2577
DDX60L	0.0010	0.7410	0.8970	**0.0031**	0.1121	**0.0394**	0.6580
EIF1AY	5.1E-11	**9.5E-14**	0.9980	0.8802	0.3737	0.4254	0.6960
FAM168A	0.0095	0.7670	0.8970	**0.0043**	0.6073	**0.0133**	0.5456
FAM49B	0.0377	0.6380	0.3550	0.5656	0.0619	0.9282	0.1931
FLJ27255	0.0144	0.4290	0.8970	0.3942	0.1404	0.0722	**0.0102**
GGCX	0.0270	0.5530	0.8970	**0.0003**	**0.0047**	**0.0011**	**0.0019**
HCAR2	0.0041	0.2320	0.8970	0.3975	0.0846	0.0943	**0.0048**
KDM5D	8.9E-06	**6.5E-09**	0.9980	0.8802	0.4820	0.6138	0.6580
KIAA0040	0.008	0.544	0.2320	0.6094	**0.0301**	0.1447	**0.0206**
LIN7A	0.000	0.736	0.8970	0.1649	0.1194	**0.0251**	**0.0028**
LINC00278	0.0004	**3.7E-07**	0.9770	0.6229	0.6583	0.9282	0.3155
LOC100506115	0.0351	0.5580	0.8970	0.3766	0.1199	0.2488	**0.0296**
LOC100507417	0.0156	0.5690	0.9980	0.6229	0.2098	0.1774	**0.0027**
LOC100996314	0.0054	0.5530	0.8970	0.2556	0.2051	**0.0240**	**0.0126**
LOC730227	0.0270	0.2320	0.9980	0.8159	0.2247	0.0826	**0.0084**
LY96	0.0017	0.6730	0.8970	0.6229	0.1216	0.2400	**0.0053**
MCTP2	0.0173	0.2770	0.8970	**0.0043**	0.7203	**0.0133**	0.2079
MME	0.0194	0.5580	0.9960	0.5715	0.1567	0.1447	**0.0133**
NID1	0.0039	0.1420	0.8970	0.4625	0.0619	0.0535	**0.0036**
NKG7	0.0411	0.9990	0.8970	0.9376	0.6160	0.6247	**0.0165**
PDE4B	0.0351	0.5530	0.8970	0.3187	0.2921	0.0826	0.0662
PRKY	0.0002	**6.1E-06**	0.9980	0.9013	0.0730	0.2275	0.7686
PSMD2	0.0377	0.7670	0.8970	**0.0361**	0.8271	0.7473	0.7686
PTPRE	0.0209	0.7670	0.9980	0.6229	0.1584	0.2843	**0.0126**
QPCT	6.2E-06	0.7670	0.0825	0.6229	**0.0047**	0.2114	**0.0019**
RNF149	0.0215	0.8300	0.8970	0.1649	0.1718	0.1537	0.2079
RPS4Y1	9.3E-12	**7.8E-15**	0.9980	0.9376	0.9670	0.7304	0.8350
SASH3	0.0266	0.5690	0.2310	0.9376	**0.0302**	0.4254	0.0558
SIGLEC9	0.0048	0.9100	0.3100	0.6229	**0.0458**	0.1774	**0.0126**
SLED1	0.0383	0.5690	0.9980	0.3942	0.2247	0.1447	**0.0279**
SUMO1P1	0.0156	0.5690	0.8970	0.3187	0.1204	0.2995	0.0701
TGM3	0.0461	0.5580	0.9980	0.3188	0.2247	**0.0240**	**0.0030**
TMSB4Y	0.0459	**2.1E-05**	0.9980	0.9229	0.8908	0.9930	0.7686
TNFRSF1B	0.0270	0.8300	0.8970	0.1553	0.2247	0.1447	0.2079
TRIB1	0.0420	0.7670	0.8970	0.6229	0.1486	0.2114	**0.0289**
TTTY15	3.5E-07	**8.3E-11**	0.8970	0.3942	0.9670	0.6247	0.0697
TXLNG2P	6.2E-06	**1.2E-07**	0.9980	0.9013	0.9601	0.3601	0.7378
TYROBP	0.0270	0.7670	0.8970	0.4988	0.1194	0.4151	0.0697
USP9Y	6.6E-07	**1.4E-08**	0.8970	0.3942	0.1718	0.7321	0.0697
UTY	3.3E-08	**8.6E-11**	0.9980	0.9376	0.6293	0.4254	0.9668
XIST	0.0002	**1.2E-07**	0.8970	0.2067	0.2898	0.1864	0.5386
ZFY	3.5E-07	**2.6E-10**	0.9980	0.9229	0.8198	0.9714	0.7733

Supplemental Table 2. Primers and Probes used in TAC Detection System.

Pathogen	Forward	Final Conc.	Reverse	Final Conc.	Key	Probe	Final Conc.	Reference
influenza A	GAC CRA TCC TGT CAC CTC TGA C	800nM	AGG GCA TTY TGG ACA AAK CGT CTA	800nM	#	FAM-TGC AGT CCT CGC TCA CTG GGC ACG-BHQ1	200nM	CDC[23], Biosearch Technologies[24]
influenza B	TCC TCA AYT CAC TCT TCG AGC G	800nM	CGG TGC TCT TGA CCA AAT TGG	800nM	#	FAM-CCA ATT CGA GCA GCT GAA ACT GCG GTG-BHQ1	200nM	CDC[23], Biosearch Technologies[24]
RSV	GGC AAA TAT GGA AAC ATA CGT GAA	500nM	TCT TTT TCT AGG ACA TTG TAY TGA ACA G	250nM	#	FAM-CTG TGT ATG TGG AGC CTT CGT GAA GCT-BHQ1	50nM	Fry, A.M.[25] , Kodani, M.[9]
PIV 1	ACA AGT TGT CAA YGT CTT AAT TCR TAT	500nM	TCG GCA CCT AAG TAR TTY TGA GTT	500nM	**	FAM-ATA GGC CAA AGA “T”TG TTG TCG AGA CTA TTC CAA	50nM	Weinberg, G. A.[26]
PIV 2	GCA TTT CCA ATC TAC AGG ACT ATG A	750nM	ACC TCC TGG TAT AGC AGT GAC TGA AC	750nM	**	FAM-CCA TTT ACC “T”AA GTG ATG GAA TCA ATC GCA AA	50nM	Kodani, M.[9]
PIV 3	TGG YTC AAT CTC AAC AAC AAG ATT TAA G	750nM	TAC CCG AGA AAT ATT ATT TTG CC	500nM	**	FAM-CCC RTC TG“T” TGG ACC AGG GAT ATA CTA CAA A	200nM	Kodani, M.[9]
hRV	CY×**<u>A</u>** GCC **<u>T</u>**GC GTG GY	1000nM	GAA ACA CGG ACA CCC AAA GTA	1000nM	× #	FAM-TCC TCC GGC CCC TGA ATG YGG C-BHQ1	100nM	Harvey, JJ.[27]
EV	GGT GGC TGC GTT GGC	1000nM	GAA ACA CGG ACA CCC AAA GTA	1000nM		FAM-TCC TCC GGC CCC TGA ATG YGG C-BHQ1	100nM	Harvey, JJ. [27]
adenovirus	GCC CCA GTG GTC TTA CAT GCA CAT C	500nM	GCC ACG GTG GGG TTT CTA AAC TT	500nM	#	FAM-TGC ACC AGA CCC GGG CTC AGG TAC TCC GA-BHQ1	100nM	Heim, A.[28], Kodani, M.[9]
coronavirus 1 (229E)	CAG TCA AAT GGG CTG ATG CA	750nM	AAA GGG CTA TAA AGA GAA TAA GGT ATT CT	500nM	#	FAM-CCC TGA CGA CCA CGT TGT GGT TCA-BHQ1	50nM	Dare, R. K.[29]
coronavirus 2 (NL63)	GAC CAA AGC ACT GAA TAA CAT TTT CC	250nM	ACC TAA TAA GCC TCT TTC TCA ACC C	250nM	**	FAM-AAC ACG CT"T" CCA ACG AGG TTT CTT CAA CTG AG	50nM	Dare, R. K.[29]
coronavirus 3 (OC43)	CGA TGA GGC TAT TCC GAC TAG GT	500nM	CCT TCC TGA GCC TTC AAT ATA GTA ACC	750nM	#	FAM-TCC GCC TGG CAC GGT ACT CCC T-BHQ1	50nM	Dare, R. K.[29]
coronavirus 4 (HKU1)	CCT TGC GAA TGA ATG TGC T	100nM	TTG CAT CAC CAC TGC TAG TAC CAC	750nM	#	FAM-TGT GTG GCG GTT GCT ATT ATG TTA AGC CTG-BHQ1	50nM	Dare, R. K.[29]
RNP3	CCA AGT GTG AGG GCT GAA AAG	600nM	TGT TGT GGC TGA TGA ACT ATA AAA GG	600nM	#	FAM-CC CCA GTC TCT GTC AGC ACT CCC TTC-BHQ1	200nM	Weinberg, G. A.[26]
GAPDH	Life Technologies							
hMPV	CAA GTG TGA CAT TGC TGA YCT RAA	600nM	ACT GCC GCA CAA CAT TTA GRA A	600nM	#	FAM-TGG CYG TYA GCT TCA GTC AAT TCA ACA GA-BHQ1	100nM	Kodani, M.[9]

hBoV	TGC AGA CAA CGC YTA GTT GTT T	500nM	CTG TCC CGC CCA AGA TAC A	500nM	#	FAM-CCA GGA TTG GGT GGA ACC TGC AAA-BHQ1	100nM	Lu, X.[30]
hPeV	GTA ACA SWW GCC TCT GGG SCC AAA AG	400nM	GGC CCC WGR TCA GAT CCA YAG T	400nM	#	FAM-CCT RYG GGT ACC TYC WGG GCA TCC TTC-BHQ1	200nM	Nix, W. A.[31], Kodani, M.[9]
H. influenzae	ATG GCG GGA ACA TCA ATG A	300nM	ACG CAT AGG AGG GAA ATG GTT	300nM	◊	FAM-CGG TAA TTG GGA TCC AT-MGB	100nM	Meyler, K. L.[32]
S. pneumoniae	ACG CAA TCT AGC AGA TGA AGC A	500nM	TCG TGC GTT TTA ATT CCA GCT	500nM	#	FAM-TGC CGA AAA CGC TTG ATA CAG GGA G-BHQ1	100nM	Carvalho, Mda. G.[33], Kodani, M.[9]
M. pneumonia	TTT GGT AGC TGG TTA CGG GAA T	500nM	GGT CGG CAC GAA TTT CAT ATA AG	500nM	#	FAM-TGT ACC AGA GCA CCC CAG AAG GGC T- BHQ1	100nM	Winchell, J. M.[34], Kodani, M.[9]
C. pneumoniae	GGG CTA TAA AGG CGT TGC TTT	500nM	AGA CTT TGT TCC AGT AGC TGT TGC T	500nM	#	FAM-CC TTG CCA ACA GAC GCT GGC G-BHQ1	100nM	Mitchell, S. L.[35], Kodani, M.[9]
M. hominis	TCA CTA AAC CGG GTA TTT TCT AAC AA	300nM	TTG GCA TAT ATT GCG ATA GTG CTT	300nM	#	FAM-CTA CCA ATA ATT TTA ATA TCT GTC GGT ATG-BHQ1	200nM	Ferandon, C. [36]
Ureaplasma	CATACAGAA GGTGCTGGT GG	500nM	CTTAGGATT TAAGTGGTG ACATAC	500nM	#	FAM-AGC TTC TAC AAA CCC AAC TAT TCC-BHQ1	400nM	Based upon Xiao, L[37], Yi, J[38]
*B. pertussis (*target I)	CAA GGC CGA ACG CTT CAT	300nM	GAG TTC TGG TAG GTG TGA GCG TAA	300nM	#	FAM-CAG TCG GCC TTG CGT GAG TGG G-BHQ1	300nM	Tatti, K. M.[39], Kodani, M.[9]
Bordetella pertussis (target II)	CGC CAG CTC GTA CTT C	700nM	GAT ACG GCC GGC ATT	700nM	#	FAM-AAT ACG TCG ACA CTT ATG GCG A-BHQ1	300nM	Tatti, K. M.[39], Kodani , M.[9]

Abbreviations: conc. =concentration

Underlining and boldface indicate a locked nucleic acid (LNA) (Exiqon, Woburn, MA)

× **Y**=mix of C and T (pyrimidine) nucleosides, similar to "P" as listed in Harvey, JJ[27] (P is a universal base; (P)=dP-CE (pyrimidine derivative), designed to base pair with either A or G)

5'FAM 3'BHQ1

" " Quotation marks around a letter indicate an internal quencher

** Internally labeled probes:5'FAM "T"=BHQ1-dT 3'=phosphorylated

◊ 5'FAM 3'MGB

Supplemental Figure Legends

Supplemental Figure 1. An illustration of the non-specific filtering method. Shown here is a histogram and estimated sample density curve of $L_i = \log_2(M_i + 1)$, the log-transformation of M_i, which is the 95% quantile of the *i*th gene expression. The red line represents the defined detection level (c=3.0 for L_i, or 7.0 for M_i) used in non-specific filtering. Among the 25,237 genes assessed, 13,978 genes passed this non-specific filtering threshold and were considered expressed in the HINT.

Supplemental Figure 2. Pathway analysis of genes with highest variation in the HINT. Pathway analysis was performed for the 200 genes identified as having the highest coefficient of variation in expression (see Methods) in the HINT, using Ingenuity Pathway Analysis (IPA) software, as described in Methods. Shown here are significantly affected canonical pathways, listing the percentage of virus-associated genes (blue bar) and the percentage of all genes in the genome that correspond to each listed pathway (red bar). The pathways are ranked by p-value ($p<0.05$), with the most significant at the top.

Supplemental Figure 3. Pathway analysis of genes associated with the presence of pathogenic virus. Pathway analysis was performed for the 282 genes identified as having significant differences in mean expression in subjects with the presence of any virus, using Ingenuity Pathway Analysis (IPA) software, as described in Methods. Shown here are significantly affected canonical pathways, listing the percentage of virus-associated genes (blue bar) and the percentage of all genes in the genome that correspond to each listed pathway (red bar). The pathways are ranked by p-value ($p<0.05$), with the most significant at the top.

Supplemental Figure 4. Pathway analysis of genes associated with Moraxella burden. Pathway analysis was performed for the 739 genes whose expression was significantly correlated with the burden of genus *Moraxella*, using Ingenuity Pathway Analysis (IPA) software, as described in Methods. Shown here are significantly affected canonical pathways, listing the percentage of Moraxella-associated genes (blue bar) and the percentage of all genes in the genome that correspond to each listed pathway (red bar). The pathways are ranked by p-value ($p<0.05$), with the most significant at the top.

Supplemental Figure 1

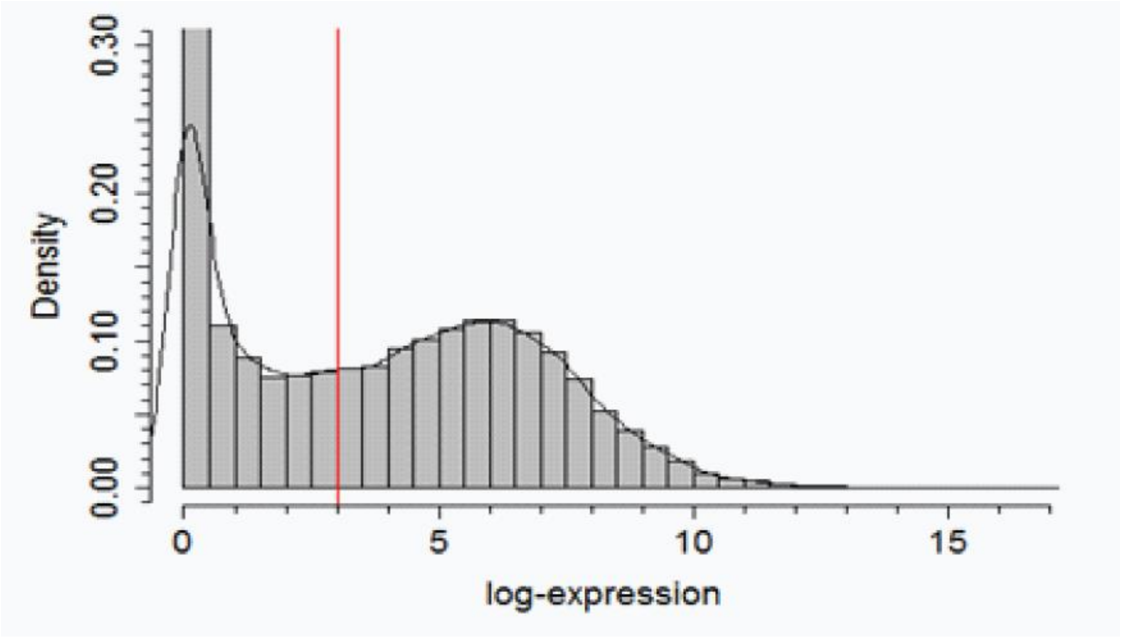

Supplemental Figure 2

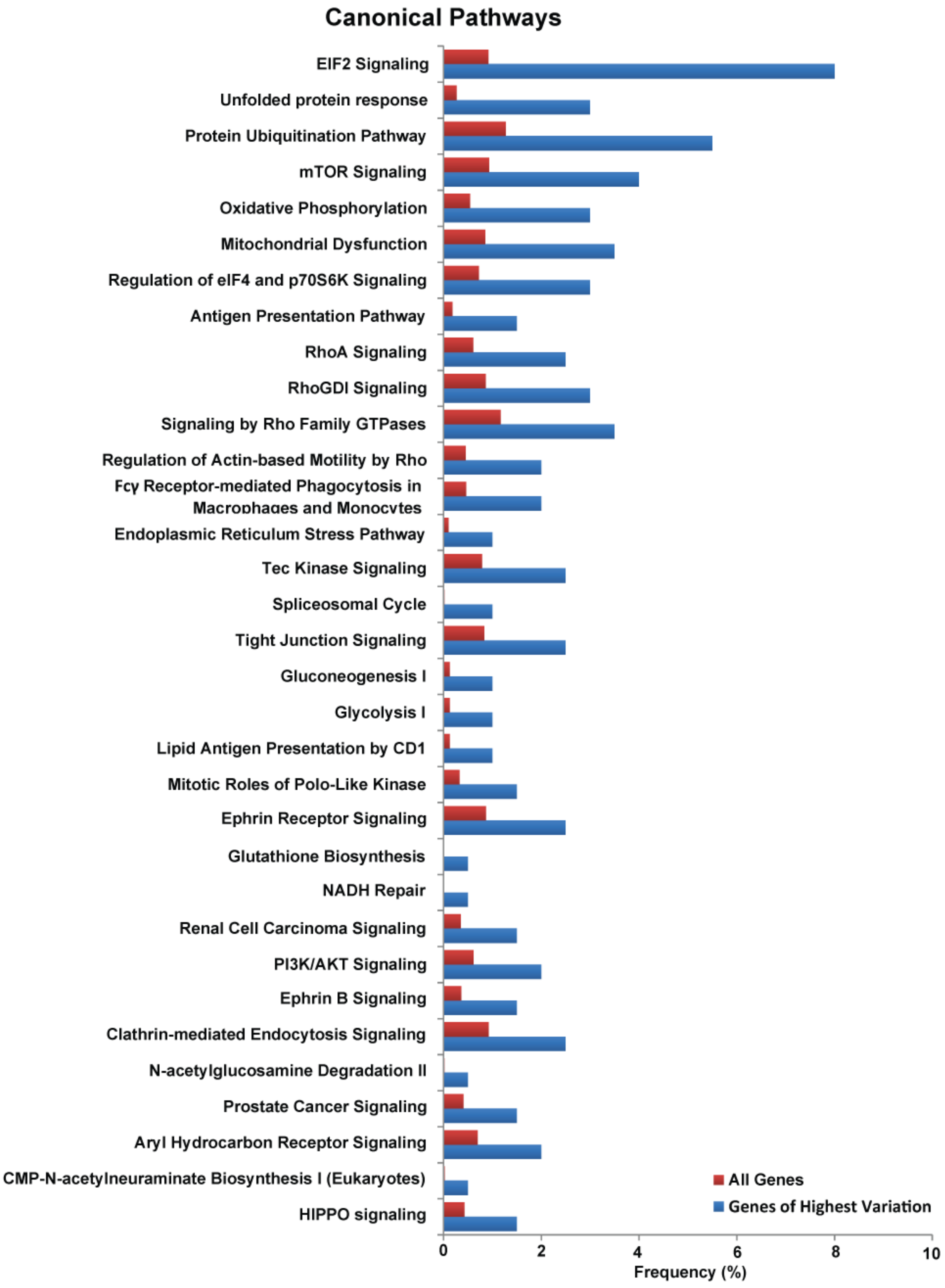

Supplemental Figure 3

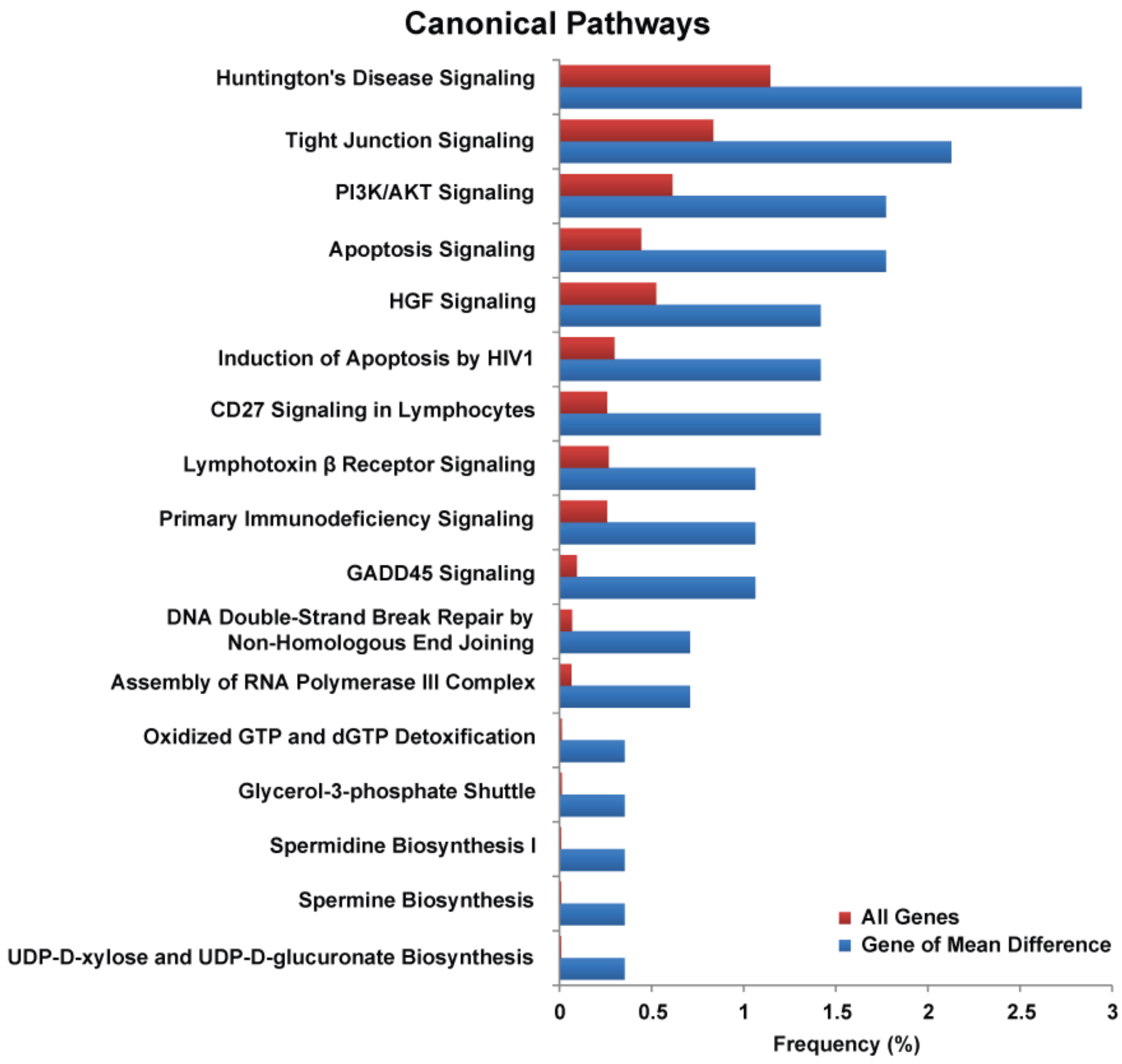

Supplemental Figure 4

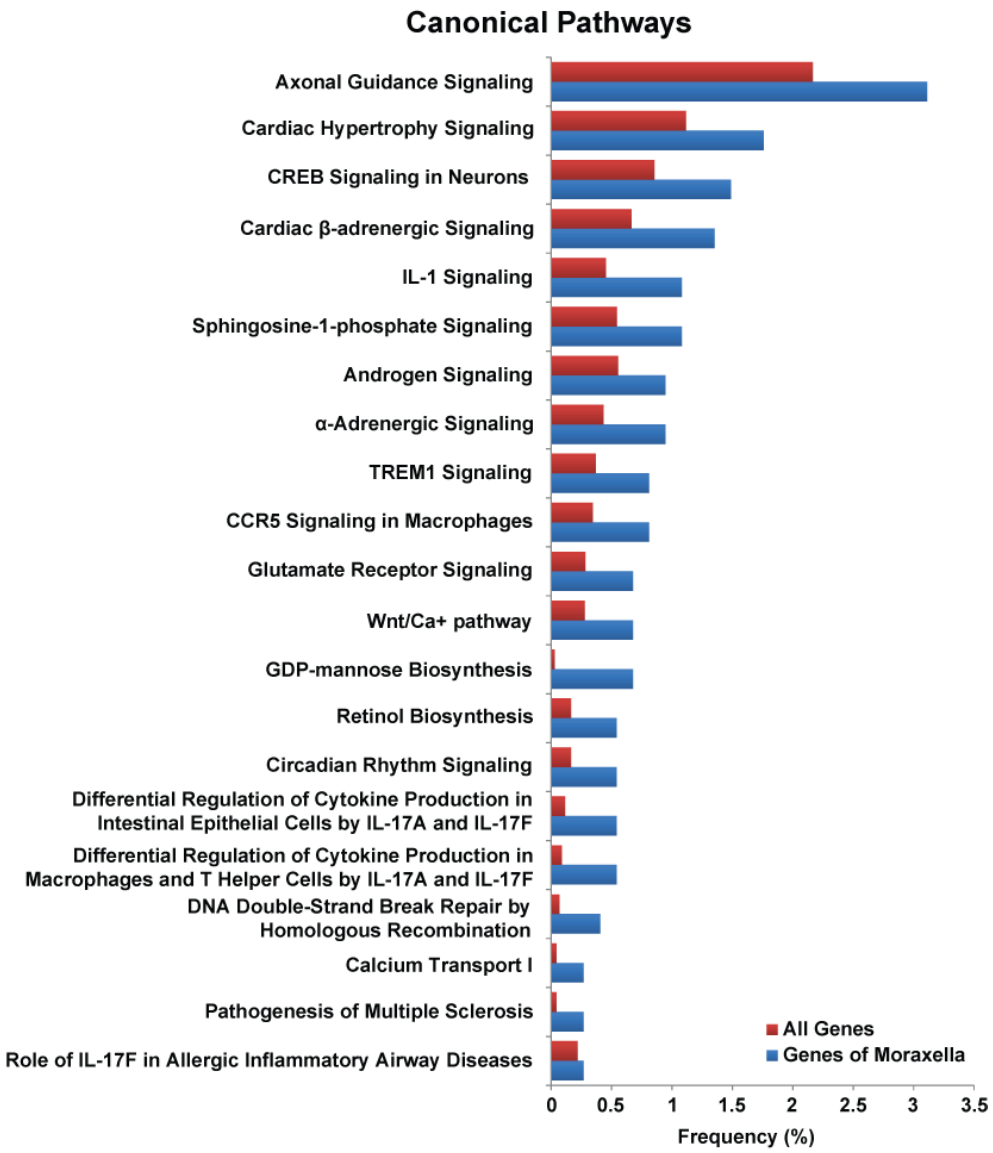

Supplement: Supplementary Information [file srep33994-s1.pdf]
